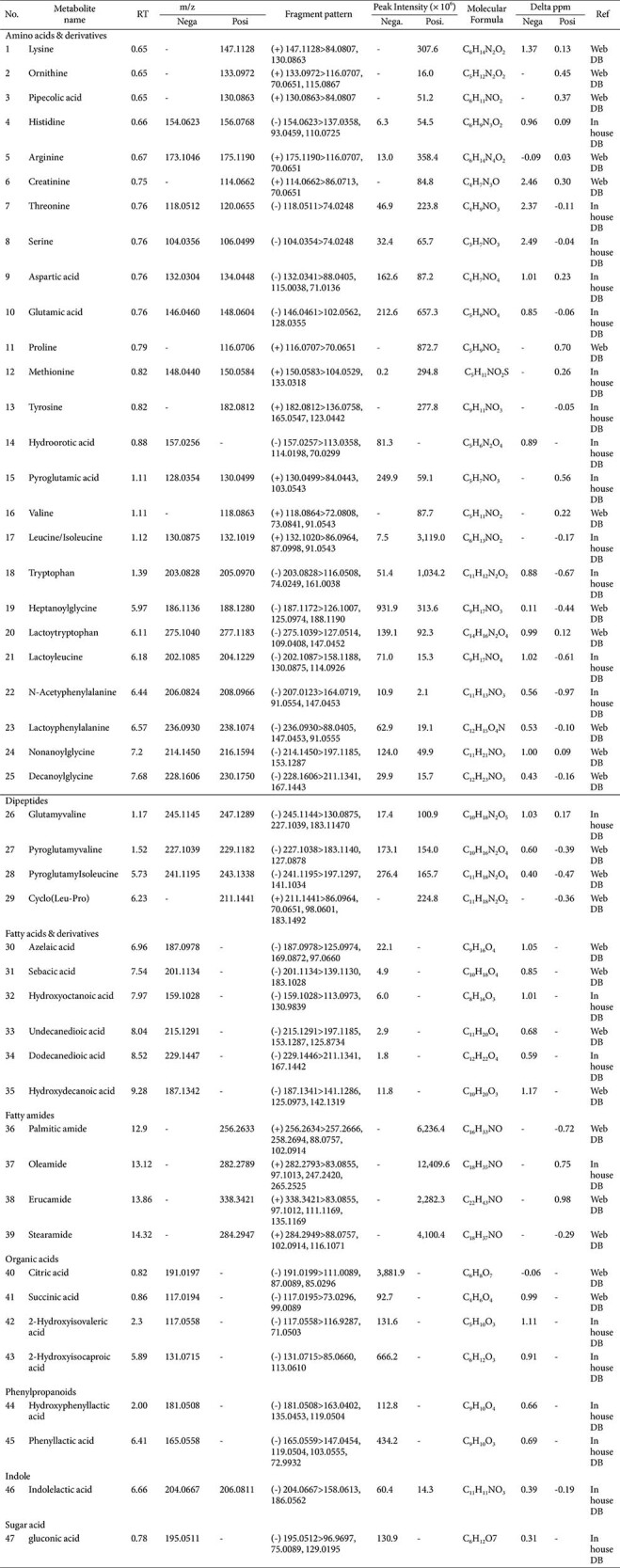# Mitigation of *Bifidobacterium longum* Z1 on Methylglyoxal-Induced Glycotoxicity in Neuron-2A Cells by Enhancing Detoxification Pathways

**DOI:** 10.4014/jmb.2504.04018

**Published:** 2025-09-11

**Authors:** Seong-Min Hong, Su-Hyun Kim, Jae-Hyuk Lee, Choong-Hwan Lee, Dong-Hyun Kim, Sun Yeou Kim

**Affiliations:** 1 College of Pharmacy and Gachon Institute of Pharmaceutical Sciences, Gachon University, Incheon 21936, Republic of Korea; 2 Division of Food Science and Technology, Gyeongsang National University, Jinju, Gyeongnam 52828, Republic of Korea; 3 Department of Bioscience and Biotechnology, Konkuk University, Seoul 05029, Republic of Korea; 4 AmtixBio Co., Ltd., Hanam-si 12925 Republic of Korea; 5 Research Institute for Bioactive-Metabolome Network, Konkuk University, Seoul 05029, Republic of Korea; 6 Neurobiota Research Center, College of Pharmacy, Kyung Hee University, Seoul 02447, Republic of Korea

**Keywords:** *Bifidobacterium longum* Z1, methylglyoxal, neuroprotective effect, N2a cells, tryptophan

## Abstract

Methylglyoxal (MGO), a reactive byproduct of microbial metabolism, contributes to neurodegeneration and may be further exacerbated by gut dysbiosis. Probiotic strategies that restore microbial balance and barrier integrity thus represent a promising therapeutic approach. In this study, we explored the neuroprotective potential of the probiotic strain *Bifidobacterium longum* Z1 (*B. longum* Z1) in MGO-challenged mouse neuronal cells (N2a). *B. longum* Z1 treatment effectively reduced apoptosis and reactive oxygen species (ROS) production, indicating its substantial neuroprotective activity. Mechanistically, *B. longum* Z1 treatment downregulated apoptotic signaling pathways involving mitogen-activated protein kinases (MAPKs) and nuclear factor kappa-light-chain-enhancer of activated B cells (NF-κB). Furthermore, *B. longum* Z1 enhanced cellular detoxification through activation of the glyoxalase system and bolstered antioxidant defenses *via* induction of nuclear factor erythroid 2-related factor 2 (Nrf2) and heme oxygenase-1 (HO-1). Metabolite analysis identified bioactive compounds within *B. longum* Z1, notably tryptophan, which exhibited a high affinity for MGO and modulated the expression of apoptosis-related proteins. Our findings indicate that *B. longum* Z1 and its microbial bioactive metabolites, including tryptophan, may serve as potential nutraceutical candidates for neuroprotection against glycotoxins such as MGO.

## Introduction

Neurodegenerative diseases (NDs), including Alzheimer’s disease (AD) and Parkinson’s disease (PD), represent significant global health challenges characterized by progressive neuronal loss, cognitive decline, motor dysfunction, and severe impairment of patient quality of life [1]. These multifaceted diseases arise from a combination of genetic susceptibility, environmental factors, metabolic disturbances, oxidative stress, chronic inflammation, and disruptions in the gut-brain axis [2]. Recent studies have underscored the strong association between gut microbiota dysbiosis and NDs, highlighting that imbalanced microbial communities can exacerbate neurodegeneration through neuroinflammation, disruption of neurotransmitter homeostasis, and compromised intestinal barrier integrity [3]. Consequently, therapeutic approaches targeting the composition and functionality of gut microbiota have gained substantial attention for preserving neuronal health and managing neurodegenerative conditions.

Additionally, chronic hyperglycemia has been closely linked to an increased risk of developing neurodegenerative diseases, highlighting the metabolic disturbances induced by hyperglycemia as critical contributors to neurodegeneration [4]. Hyperglycemia promotes gut microbiota dysbiosis, resulting in the production of highly reactive metabolites, notably methylglyoxal (MGO), a reactive carbonyl species (RCS) [5]. MGO is implicated in various diabetic complications, including cardiovascular diseases and neurodegenerative disorders, such as AD and PD, primarily through the formation of advanced glycation end products (AGEs) [6]. Several studies have reported that AGE accumulation in the brain significantly exacerbates AD pathology [7]. Elevated AGE levels serve as pathological markers in patients with diabetic complications, indicating an increased vulnerability to AD progression [8]. Previous studies have substantiated the role of MGO in cognitive impairment and depression-like behaviors, thereby underscoring its importance as a pathological mediator of AD [9].

Currently, no drugs effectively halt AD progression, and existing pharmacological treatments provide only symptomatic relief [10]. Recent studies have emphasized that dietary supplements with neuroprotective properties are promising functional food ingredients, particularly for elderly individuals seeking to prevent or slow cognitive decline [10]. Numerous clinical studies and systematic reviews have highlighted the significant role of dietary supplements, including vitamins, minerals, antioxidants, and polyunsaturated fatty acids, in maintaining and improving cognitive function in older adults. These supplements have been shown to support memory, attention, executive function, and provide neuroprotection, with some demonstrating the potential to delay the onset or progression of cognitive decline and dementia. As a result, global awareness regarding the use of nutritional strategies as a non-pharmacological approach to preserve cognitive health is growing, especially in aging populations [11].

Probiotics, live microorganisms that provide health benefits when consumed in adequate amounts, have shown promising effects on human health. Specific probiotic strains, particularly *Lactobacillus* and *Bifidobacterium* species, have demonstrated beneficial effects on intestinal health, immune modulation, obesity, and cancer prevention [12, 13]. Recent evidence also demonstrates their capacity to influence central nervous system function through the gut-brain axis [14]. Animal studies have revealed that *Lactobacillus* and *Bifidobacterium* species alleviate anxiety-like behaviors and modulate neuronal functions, potentially enhancing memory-related neurotransmitters such as acetylcholine and glutamate, and increasing the expression of neurotrophic factors such as brain-derived neurotrophic factor (BDNF) and synapsin [12, 13]. Furthermore, probiotic mixtures including *Lactobacillus* and *Bifidobacterium* species have demonstrated therapeutic potential in managing neurodegenerative disorders [15]. Collectively, these findings suggest that probiotics may offer therapeutic or preventive benefits against AD-associated cognitive impairment.

In a preliminary screen, *Bifidobacterium* species exhibited inhibitory effects against MGO and its derived advanced glycation end products (MGO-AGEs). Among these species, *Bifidobacterium longum* Z1 strain emerged as a particularly promising candidate. This study investigated the neuroprotective potential of *B. longum* Z1 against MGO-induced toxicity in neuroblastoma N2a cells, focusing specifically on mechanisms related to oxidative stress, apoptosis, inflammation, glyoxalase detoxification, and the antioxidant defense system.

## Materials and Methods

### Materials and Reagents

N2a cells were obtained from American Type Culture Collection (ATCC CCL-131, USA). MGO and tryptophan (Trp) were purchased from Sigma-Aldrich (USA). Dulbecco’s modified Eagle’s medium (DMEM), fetal bovine serum (FBS), and penicillin-streptomycin (PS) were obtained from Gibco (USA). Primary antibodies against protein kinase B (AKT), phosphorylated AKT (p-AKT), Bax, Bcl-2, cytochrome C, caspase-3, p38, phosphorylated p38 (p-p38), c-Jun *N*-terminal kinase (JNK), phosphorylated JNK (p-JNK), extracellular signal-regulated kinase (ERK), phosphorylated ERK (p-ERK), inhibitor of nuclear factor kappa B (IκB), nuclear factor kappa-light-chain-enhancer of activated B cells (NF-κB), histamine, and α-tubulin were purchased from Cell Signaling Technology (USA). Additional primary antibodies against glyoxalase-1, glyoxalase-2, nuclear factor erythroid 2-related factor 2 (Nrf2), and heme oxygenase-1 (HO-1) were purchased from Santa Cruz Biotechnology (USA). All other chemicals and reagents were acquired from Sigma-Aldrich.

### Bacterial Strain and Cell Cultures

*B. longum* Z1 was isolated from human feces and identified by 16S rRNA gene sequencing (Table S1). The strain was anaerobically cultured in Gifu Anaerobic Medium (GAM; Nissui Pharmaceutical, Japan) at 37°C for 3 days. Cultured bacteria were harvested by centrifugation, freeze-dried, resuspended in phosphate-buffered saline (PBS, pH 7.4), and heat-killed at 80°C for 10 min. The inactivated cells were stored at −80°C until further use. For cell experiments, mouse N2a neuroblastoma cells were cultured in Dulbecco’s Modified Eagle Medium (DMEM) supplemented with 10% fetal bovine serum (FBS) and 1% penicillin–streptomycin (PS), and maintained at 37°C in a humidified incubator with 5% CO_2_. *B. longum* Z1 was administered at concentrations of 1, 5, and 10 μg/mL, corresponding to approximately 0.5 × 10^5^, 2.5 × 10^5^, and 5 × 10^5^ CFU/mL, respectively, based on dry weight conversion. These concentrations were selected to evaluate concentration-dependent effects within a non-cytotoxic range suitable for *in vitro* assays. The range was chosen based on experimental feasibility and to ensure detection of graded biological responses under MGO-induced stress conditions.

### Cell Viability

Cell viability was assessed using the MTT [(3-(4,5-dimethylthiazol-2-yl)-2,5-diphenyltetrazolium bromide)] assay. N2a cells (2 × 10^4^ cells/mL) were seeded into 96-well plates, pre-treated with *B. longum* Z1 (1, 5, and 10 μg/mL) or aminoguanidine (AG, 1 mM) for 1 h, followed by treatment with 500 μM MGO for 24 h (Fig. S1). Cells were then incubated with 0.5 mg/ml MTT solution for 1 h, the solution was removed, and 200 μl dimethyl sulfoxide (DMSO) was added. The absorbance was measured at 570 nm using a plate reader (Bio-Rad, USA).

### 5'-Dromo-2'-Deoxyuridine (BrdU) Cell Proliferation and Lactate Dehydrogenase (LDH) Assay

Cell proliferation and cytotoxicity were measured using a BrdU incorporation kit and lactate dehydrogenase (LDH) cytotoxicity detection kit (Cell Signaling Technology, USA), respectively, following the manufacturer's instructions.

### Reactive Oxygen Species (ROS) Measurement

Intracellular ROS levels were evaluated using 2',7'-dichlorodihydrofluorescein diacetate (DCFH-DA) in both a plate reader assay and confocal imaging. For quantification, N2a cells (2 × 10^4^ cells/mL) were seeded in 96-well plates, pre-treated with *B. longum* Z1 (10 μg/mL) or aminoguanidine (AG, 1 mM) for 1 h, then exposed to MGO (500 μM) for 24 h. Cells were incubated with 20 μM DCFH-DA for 30 min at 37°C, and fluorescence (Ex/Em = 485/535 nm) was measured using a VICTOR X3 plate reader (PerkinElmer, USA). For imaging, N2a cells (1 × 10^5^) were seeded in 35 mm confocal dishes, treated similarly with *B. longum* Z1 (1, 5, or 10 μg/ml) or 10 mM AG, and exposed to MGO for 2 h. After washing with PBS, cells were stained with 4',6-diamidino-2-phenylindole (DAPI, 1 μg/ml) and incubated with 20 μM DCFH-DA. Fluorescence was visualized using a Nikon A1 Plus confocal microscope and quantified with NIS-Elements software (v6.10.01).

### Metabolite Analysis

To extract the intracellular metabolites, 50 mg of *B. longum* Z1 was suspended in 1ml of pure methanol. The samples were homogenized for 10 min using a mixer mill, followed by sonication for 10 min at room temperature. After centrifugation at 15,000 ×*g* for 10 min at 4°C, the supernatant was carefully collected and concentrated using a speed vacuum. The dried extract was reconstituted in 100% methanol to achieve a final concentration of 4 mg/ml. A 100 μl aliquot was transferred into a vial for subsequent mass spectrometry analysis. Metabolite profiling was conducted using a Vanquish binary pump C system (Thermo Fisher Scientific) coupled with a Waters ACQUITY UPLC HSS T3 column (150 mm × 2.1 mm, 1.8 μm particle size; Waters) and an Orbitrap Exploris 120 mass spectrometer (Thermo Fisher Scientific), following the method described by Na *et al*., [16].

### MGO-Affinity Assay

The affinity of MGO for 13 different amino acids was analyzed as previously described, with minor modifications [9]. Briefly, reactions containing MGO with or without test samples were incubated in PBS (pH 7.4) with 0.02%sodium azide at 37°C for one week. Fluorescence intensity was measured at excitation/emission wavelengths of 355/460 nm using a multilabel plate reader (VICTOR X3, PerkinElmer, USA).

### Western Blot Assay

N2a cells were harvested and lysed using PRO-PREP protein extraction solution (iNtRON Biotechnology, Republic of Korea) supplemented with protease and phosphatase inhibitors. Cytosolic and nuclear fractions were prepared as required. Protein concentrations were determined using the Bradford assay. Equal protein amounts (30–40 μg) underwent electrophoresis, were transferred to polyvinylidene fluoride (PVDF) membranes, blocked in 5% skim milk, and incubated overnight with primary antibodies at 4°C. Following incubation with horseradish peroxidase-conjugated secondary antibodies, chemiluminescence was detected using a Bio-Rad ChemiDoc XRS+ imaging system. Bands were quantified using Image Master 2D Elite software.

### Nitric Oxide (NO) Assay and Enzyme-Linked Immunosorbent Assay (ELISA)

N2a cells (2 × 10^4^ cells/ml) were seeded in 96-well plates and pre-treated with *B. longum* Z1 (1, 5, or 10 μg/mL) or aminoguanidine (AG, 1 mM) for 30 min, followed by stimulation with lipopolysaccharide (LPS, 1 μg/mL) for 24 h [17, 18]. Cell viability was assessed by MTT assay using 0.5 mg/mL MTT for 1 h. After removing the supernatant, 200 μL of DMSO was added to dissolve formazan crystals, and absorbance was measured at 570 nm using a microplate reader (Bio-Rad). Nitric oxide production was determined in the culture supernatant using Griess reagent (Jiancheng Institute of Biotechnology, China), and absorbance was measured at 520 nm (Bio-Rad). Levels of pro-inflammatory cytokines such as interleukin (IL)-1β, IL-6, and tumor necrosis factor-α (TNF-α) were quantified using ELISA kits (R&D Systems, USA) according to the manufacturer’s instructions.

### Neurite Outgrowth Assay

Neurite outgrowth was analyzed by seeding N2a cells at 2 × 10^4^ cells/well in 24-well plates, treating the cells as indicated, and capturing images of neurite length using an IncuCyte imaging system (Essen Instruments, USA) [19].

### Statistical Analysis

Results are presented as mean ± SEM, analyzed using GraphPad Prism 5.0 software (GraphPad Software Inc., USA). Data were statistically evaluated using one-way analysis of variance (ANOVA) for multiple comparisons, Student's *t*-test for paired comparisons, and two-way ANOVA for neurite outgrowth analysis. Statistical significance was set at *p* < 0.05.

## Results

### *B. longum* Z1 Suppressed MGO-Induced Glycotoxicity in N2a Cells

**Fig. 1:**
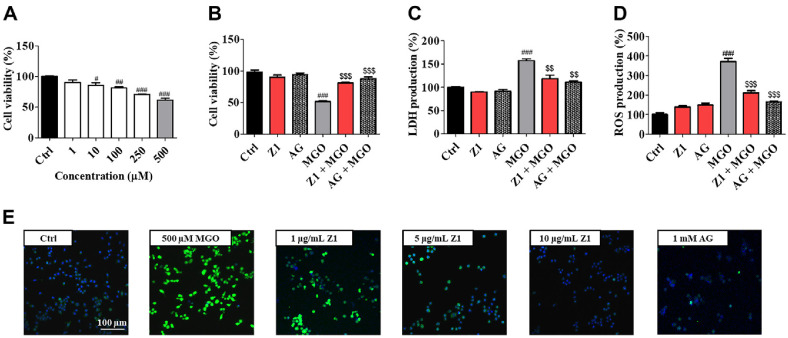
Effects of *B. longum* Z1 on methylglyoxal (MGO)-induced glucotoxicity in neuronal cells (N2a) cells. (**A**) Cell viability of N2a cells treated with various MGO concentrations (1, 10, 100, 250, and 500 μM) for 24 h. (**B–E**) Cell viability, proliferation, lactate dehydrogenase (LDH) release, and reactive oxygen species (ROS) generation in N2a cells pretreated with *B. longum* Z1 (10 μg/mL) for 1 h, followed by MGO (500 μM) exposure for 24 h. (**E**) ROS levels were evaluated following 1 h of MGO treatment. Green fluorescence (DCF-DA) indicates ROS generation detected by DCF-DA staining and visualized using the confocal microscope. Scale bar = 100 μm. Data are expressed as mean ± SEM (*n* = 3). ^#^*P* < 0.05 and ^###^*P* < 0.001 vs. Control (Ctrl) group. ^$^*P* < 0.05, ^$$^*P* < 0.01, and ^$$$^*P* < 0.001 vs. MGO-induced (MGO) group.

MGO exerts cytotoxic effects on neuronal cells in the human brain [20]. To evaluate the cytotoxic effects of MGO in neuronal N2a cells, the cells were treated with various concentrations of MGO. As shown in Fig. 1A, MGO induced significant cytotoxicity in N2a cells in a concentration-dependent manner. Among the tested concentrations, treatment with 500 μM MGO notably reduced cell viability to 61.58% ± 3.11% (^###^*P* < 0.001) compared to the control (Ctrl) group. Consequently, 500 μM MGO was used to investigate the neuroprotective effects of *B. longum* Z1 against MGO-induced toxicity in N2a cells. Treatment with *B. longum* Z1 at a concentration of 10 μg/ml significantly improved cell viability (81.02 ± 1.27%, ^###^*P* < 0.001), exhibiting efficacy comparable to the known anti-glycation agent, aminoguanidine (1 mM), which showed 87.68 ± 3.14% cell viability (^###^*P* < 0.001). Moreover, *B. longum* Z1 significantly enhanced cell viability in the presence and absence of MGO (Fig. S1). Additionally, exposure to MGO significantly LDH release (157.02 ± 3.45%, ^###^*P* < 0.001) and ROS production (370.53 ± 17.11%, ^###^*P* < 0.001). In contrast, co-treatment with *B. longum* Z1 (10 μg/ml) significantly mitigated these increases, reducing LDH levels to 111.17 ± 2.72% (^###^*P* < 0.001) and ROS levels to 164.90 ± 3.51% (^###^*P* < 0.001) (Fig. 1C and 1D). Representative ROS imaging further demonstrated the protective effect of *B. longum* Z1, confirming decreased ROS levels compared to those in MGO-treated cells (Fig. 1E). These findings indicate that *B. longum* Z1 exerts protective effects against MGO-induced glycotoxicity in neuronal N2a cells.

### *B. longum* Z1 Prevents the MGO-Induced Apoptosis Pathways in N2a Cells

**Fig. 2:**
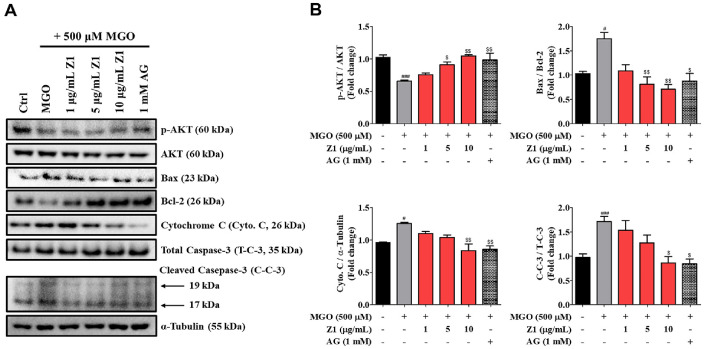
Effects of *B. longum* Z1 on MGO-induced apoptosis in N2a cells. Cells were pre-treated with *B. longum* Z1 (1, 5, and 10 μg/mL) for 1 h, followed by MGO (500 μM) for 24 h. (**A**) Western blot analysis showing expression of p-AKT/AKT, Bax/Bcl-2, cytochrome c (Cyto. C), and cleaved caspase-3 (C-C-3)/total caspase-3 (T-C-3). (**B**) Densitometric quantification of band intensities. Protein levels were normalized to α-tubulin. Data are presented as mean ± SEM (*n* = 3). ^#^*P* < 0.05 and ^###^*P* < 0.001 vs. Control (Ctrl) group. ^$^*P* < 0.05, ^$$^*P* < 0.01, and ^$$$^*P* < 0.001 vs. MGO-induced (MGO) group.

The Bcl-2 family of proteins, which act downstream of the phosphatidylinositol 3-kinase (PI3K)/AKT/mammalian target of rapamycin (mTOR) signaling pathway, are critical regulators of mitochondrial apoptosis. They control mitochondrial outer membrane permeability, cytochrome c release, and subsequent caspase-3 activation in response to various cellular stressors [21]. Among these stressors, MGO is also known to trigger apoptosis through the mechanisms described above [22]. As shown in Fig. 2, treatment with MGO significantly upregulated the expression of the pro-apoptotic protein Bax (^#^*P* < 0.05) and downregulated the anti-apoptotic protein Bcl-2 (^#^*P* < 0.05) by modulating AKT signaling (^###^*P* < 0.001). Additionally, the expression levels of cytochrome c (^#^*P* < 0.05) and cleaved caspase-3 (^###^*P* < 0.001) were significantly higher than those in the Ctrl group. Meanwhile, treatment with *B. longum* Z1 at 10 μg/mL significantly reduced the Bax/Bcl-2 expression ratio (^$$^*P* < 0.01) through modulation of AKT signaling (^$$^*P* < 0.01), and also reduced the expression levels of cytochrome c (^$$^*P* < 0.01) and cleaved caspase-3 (^$$^*P* < 0.01), displaying effects comparable to 1 mM AG (^$$^*P* < 0.001). These findings suggest that *B. longum* Z1 effectively suppresses MGO-induced apoptosis, thereby exerting a neuroprotective effect.

### *B. longum* Z1 Attenuates MGO-Induced Activation of Inflammatory Pathways in N2a Cells

**Fig. 3:**
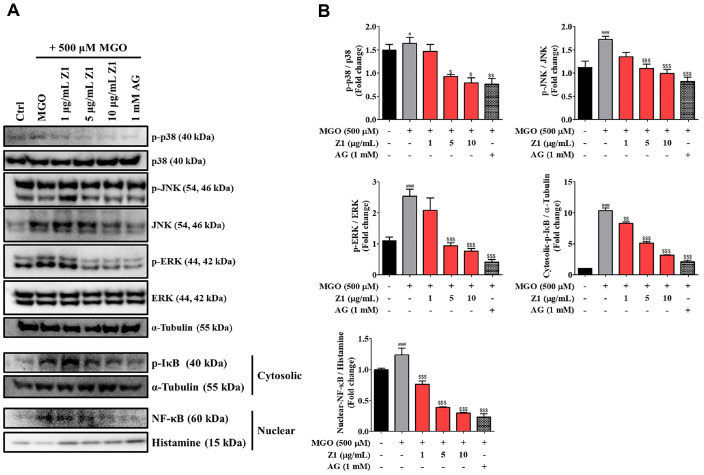
Effects of *B. longum* Z1 on MGO-induced MAPK and NF-κB signaling pathways in N2a cells. To evaluate mitogen activated protein kinase (MAPK) activation, N2a cells were pre-treated with *B. longum* Z1 (1, 5, and 10 μg/mL) for 1 h, followed by MGO (500 μM) for 30 min. For nuclear factor (NF)-κB and phosphorylated IκB (p-IκB) analysis, cells were similarly pre-treated and then exposed to MGO for 24 h. (**A**) Western blot analysis of p-p38/p38, p-JNK/JNK, p-ERK/ERK, cytosolic p-IκB, and nuclear NF-κB. (**B**) Densitometric quantification of each band. Protein levels were normalized to α- tubulin or histone. ^#^*P* < 0.05 and ^###^*P* < 0.001 vs. Control (Ctrl) group. ^$^*P* < 0.05, ^$$^*P* < 0.01, and ^$$$^*P* < 0.001 vs. MGO-induced (MGO) group.

To elucidate the mechanisms underlying the protective effects of *B. longum* Z1 against MGO-induced cytotoxicity in N2a cells, we evaluated the phosphorylation levels of MAPK signaling pathways, including p38, JNK, and ERK, as shown in Fig. 3. MGO treatment significantly elevated p38 phosphorylation levels by 9.2 % (^#^*P* < 0.05), 53.89% (^###^*P* < 0.001), and 130.40% (^###^*P* < 0.001) compared to the Ctrl group. However, cotreatment with *B. longum* Z1 reduced the phosphorylation of these MAPKs in a concentration-dependent manner. Notably, treatment with *B. longum* Z1 at 10 μg/ml markedly inhibited the phosphorylation of p38, JNK, and ERK to 58.62%(^$$$^*P* < 0.001), 80.76% (^$$$^*P* < 0.001), and 192.91% (^$$$^*P* < 0.001) relative to the MGO-treated group, respectively. These findings suggest that the inhibition of MAPK signaling pathways contributes significantly to the neuroprotective effects of *B. longum* Z1 against MGO-induced toxicity in N2a cells. It is well-known that the nuclear translocation of NF-κB is triggered by the phosphorylation and subsequent degradation of IκB [23]. Therefore, we investigated whether NF-κB activation, which modulates inflammatory responses, was involved in the protective effects of *B. longum* Z1 in N2a cells. Notably, treatment with *B. longum* Z1 at high doses, such as 10 μg/mL, significantly reduced the nuclear translocation of NF-κB (^$$$^*P* < 0.001) and decreased the cytosolic degradation of IκB (^$$$^*P* < 0.001). These findings indicate that *B. longum* Z1 effectively inhibits MGO-induced NF-κB activation by regulating IκB expression, contributing to its anti-inflammatory and neuroprotective properties.

**Fig. 4:**
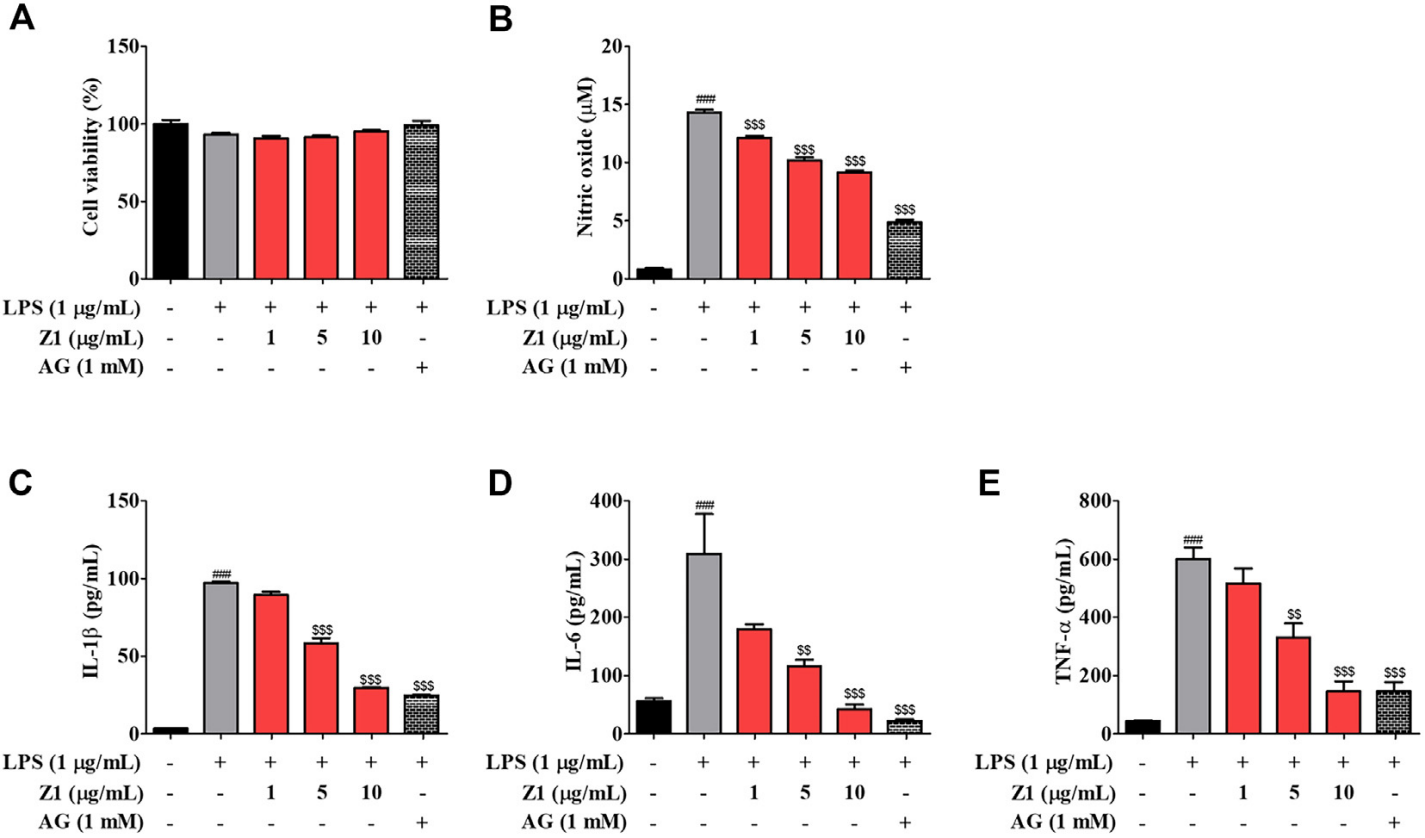
Effects of *B. longum* Z1 on lipopolysaccharide (LPS)-stimulated nitric oxide (NO) production and inflammatory cytokine secretion in N2a cells. (**A**) Cell viability in N2a cells pre-treated with *B. longum* Z1 (1, 5, and 10 μg/mL) for 1 h, followed by LPS (1 μg/mL) exposure for 24 h. (**B**) NO production in the culture supernatant was determined using the Griess reagent. (**C**–**E**) Pro-inflammatory cytokines IL-1β, IL-6, and TNF-α were quantified in the medium using ELISA kits. ^###^*P* < 0.001 vs. Control (Ctrl) group. ^$$^*P* < 0.01 and ^$$$^*P* < 0.001 vs. LPS-induced (LPS) group.

*B. longum* Z1 Attenuates LPS-Stimulated NO Production and Inflammatory Cytokine Secretion in N2a Cells To investigate the anti-inflammatory effects of *B. longum* Z1, nitric oxide and cytokine levels were measured in LPS-stimulated N2a cells, as MGO did not elicit inflammatory mediator production under the same conditions (data not shown). As shown in Fig. 4, LPS treatment significantly increased (14.30 ± 0.25 μM ^###^*P* < 0.001) NO production compared to the control group (0.82 ± 0.09 μM). However, pretreatment with *B. longum* Z1 at concentrations of 1 (12.14 ± 0.17 μM, ^$$$^*P* < 0.001), 5 (12.13 ± 0.17 μM, ^$$$^*P* < 0.001), and 10 μg/mL (10.19 ± 0.26 μM, ^$$$^*P* < 0.001) markedly reduced NO levels in a dose-dependent manner, comparable to the AG-treated positive control (4.88 ± 0.21 μM, ^$$$^*P* < 0.001). Similarly, LPS stimulation elevated the levels of pro-inflammatory cytokines IL-1β, IL-6, and TNF-α. Pre-treatment with *B. longum* Z1 significantly suppressed the expression of these cytokines in a concentration-dependent fashion. Notably, 10 μg/ml of *B. longum* Z1 (IL-1β, 29.51 ± 0.51 pg/ml; IL-6, 42.04 ± 8.56 pg/ml; TNF-α, 145.80 ± 34.00 pg/ml) exhibited the strongest inhibitory effect, closely matching that of AG (IL-1β, 24.80 ± 0.21 pg/mL; IL-6, 21.82 ± 3.21 pg/mL; TNF-α, 145.99 ± 31.18 pg/mL). These results suggest that *B. longum* Z1 effectively attenuates LPS-induced neuroinflammatory responses by downregulating NO and pro-inflammatory cytokine production in N2a cells.

### *B. longum* Z1 Promotes MGO Detoxification and Upregulates Antioxidant Defense Pathways in N2a Cells

**Fig. 5:**
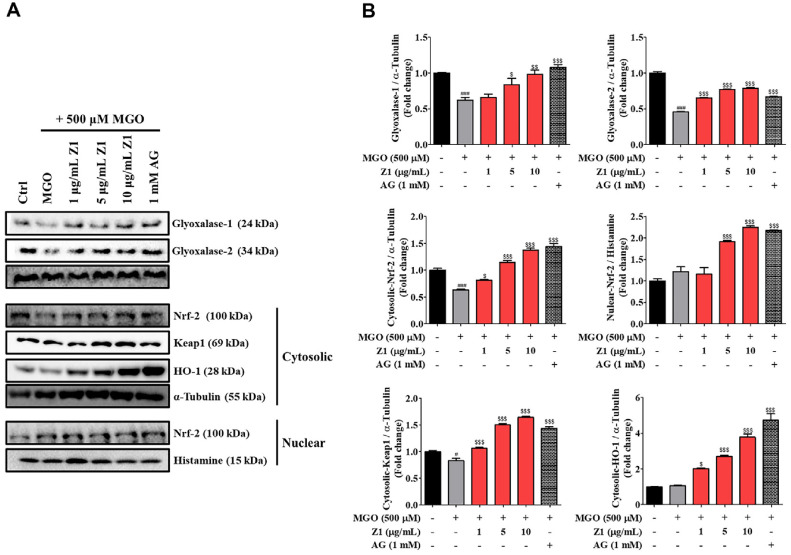
Effects of *B. longum* Z1 on MGO-induced glyoxalase and antioxidant defense pathways in N2a cells. Cells were pre-treated with *B. longum* Z1 (1, 5, and 10 μg/mL) for 1 h, followed by MGO (500 μM) for 24 h.(**A**) Western blot analysis of glyoxalase-1, glyoxalase-2, cytosolic and nuclear Nrf2, cytosolic Keap1, and HO-1. (**B**) Densitometric analysis of protein bands, normalized to α-tubulin or histone. Data are presented as mean ± SEM (*n* = 3). ^#^*P* < 0.05 and ^###^*P* < 0.001 vs. Control (Ctrl) group. ^$^*P* < 0.05, ^$$^*P* < 0.01, and ^$$$^*P* < 0.001 vs. MGO-induced (MGO) group.

MGO is detoxified through the glyoxalase system, where it is converted into d-lactate using glutathione as a cofactor, primarily *via* the sequential action of glyoxalase 1 and glyoxalase 2 [24, 25]. In addition, the Nrf2/Keap1/HO-1 signaling pathway mediates the regulation of both inflammatory and antioxidant responses [26]. To evaluate the effects of *B. longum* Z1 on the glyoxalase and antioxidant defense system, western blotting was performed (Fig. 5). MGO treatment significantly reduced the glyoxalase -1 and -2 protein levels to 34.16% (^$$$^*P* < 0.001) and 34.72% (^$$$^*P* < 0.001), respectively, compared to the control levels. In contrast, treatment with *B. longum* Z1 significantly increased the expression of both the enzymes in a concentration-dependent manner. Notably, treatment with *B. longum* Z1 at 10 μg/mL markedly elevated the glyoxalase-1 and -2 levels to 45.80% (^$$$^*P* < 0.001) and 21.27% (^$$$^*P* < 0.001), respectively. Furthermore, MGO exposure alone significantly reduced the expression of cytocylic-Nrf2 (^###^*P* < 0.001), -Keap1(^###^*P* < 0.001), and -HO-1 (^###^*P* < 0.001) compared with the control (Con) group. In contrast, treatment with *B. longum* Z1 significantly upregulated the expression of Nrf2/Keap1/HO-1 in a concentration-dependent manner in MGO-induced N2A cells. Notably, treatment with *B. longum* Z1 at 10 μg/mL markedly increased the expression of glyoxalase-related enzymes (^$$$^*P* < 0.001) and antioxidant defense system factors (^$$$^*P* < 0.001). These findings suggest that *B. longum* Z1 may confer protective effects against MGO-induced cytotoxicity and inflammation by modulating the glyoxalase system and Nrf2/Keap1/HO-1 signaling pathway.

### Tryptophan (Trp) Metabolites Identified from *B. longum* Z1 Contribute to Its Neuroprotective Effects

**Fig. 6:**
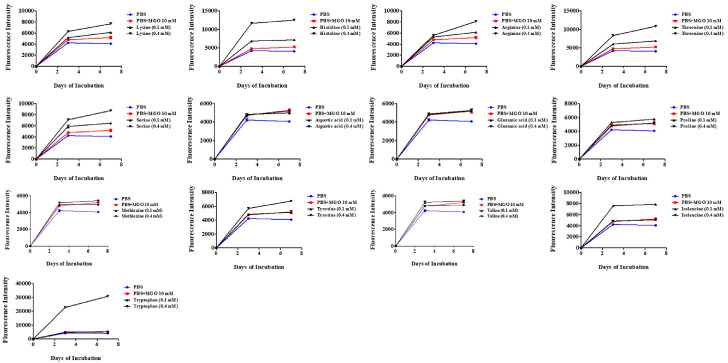
Effects of 13 amino acids on MGO-binding affinity. Fluorescence intensity was measured after co-incubating MGO (10 mM) with amino acids (100 and 400 μM), using excitation/emission wavelengths of 355/460 nm. All values were normalized to phosphate buffered saline (PBS) controls.

Metabolite profiling of *B. longum* Z1 was conducted using ultra-high-performance liquid chromatography-linear ion trap-Orbitrap hybrid mass spectrometry (UHPLC-LTQ-Orbitrap-MS/MS) to identify the bioactive compounds that potentially contribute to its neuroprotective effects (Table 1). Untargeted metabolomics is an unbiased approach used to characterize the comprehensive metabolic profiles of small molecules in biological systems such as plants, microbes, and fermented foods using high-throughput mass spectrometry [27]. In our analysis, 47 potential marker compounds were identified, including 25 amino acids and their derivatives, four dipeptides, six fatty acids and derivatives, four fatty amides, four organic acids, and other minor components. Notable compounds included amino acids such as Trp and isoleucine, and aromatic amino acid catabolites such as phenyllactic acid and hydroxyphenyllactic acid. Our previous study showed that Trp deficiency is linked to MGO-induced brain dysfunction, suggesting that Trp derived from *B. longum* Z1 may contribute to neuroprotective effects in neuronal cells [9]. Based on these findings, we focused on amino acids to evaluate their potential biological roles. As shown in Fig. 6, we evaluated the reactivity between MGO and 13 amino acids abundant in *B. longum* Z1 to assess their anti-glycotoxin potential. Remarkably, Trp exhibited the highest affinity for MGO, indicating a strong glycotoxin scavenging effect. These results support the hypothesis that Trp derived from *B. longum* Z1 modulates MGO levels, potentially contributing to the prevention of neurodegenerative conditions. Therefore, we investigated the potential effects of Trp on MGO-induced cytotoxicity in N2a cells.

**Fig. 7:**
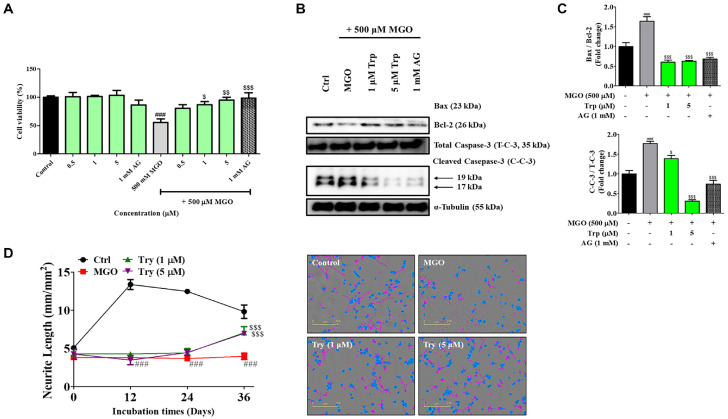
Neuroprotective effects of tryptophan (Trp) against MGO-induced toxicity in N2a cells. Cells were treated with Trp in the presence or absence of MGO (500 μM) for 24 h. (**A**) Cell viability following Trp treatment. (**B**) Expression of apoptosis-related proteins: p-AKT/AKT, Bax/Bcl-2, cytochrome c, and cleaved caspase-3 (C-C-3)/total caspase-3 (T-C-3). (**C**) Densitometric quantification of protein bands. (**D**) Neurite outgrowth was monitored for 36 h and visualized at the endpoint (scale bar = 200 μm). Purple lines indicate neurite extensions. Neurite length was quantified using imaging software. Protein levels were normalized to α- tubulin. Data are presented as mean ± SEM (*n* = 3). ^###^*P* < 0.001 vs. Control (Ctrl) group. ^$^*P* < 0.05, ^$$^*P* < 0.01, and ^$$$^*P* < 0.001 vs. MGO-induced (MGO) group.

We explored the cytoprotective effects of Trp on MGO-treated N2a cells, as shown in Fig. 7A. Trp significantly increased cell viability in a concentration-dependent manner, similar to the effects of aminoguanidine (1 mM AG). Additionally, treatment with Trp at 5 μM significantly reduced the Bax/Bcl-2 expression ratio (^$$$^*P* < 0.001) and cleaved caspase-3 levels (^$$$^*P* < 0.001), comparable to the effects of aminoguanidine (1 mM AG, ^$$$^*P* < 0.001)(Fig. 7B and 7C). Interestingly, MGO-treated cells (3.38 ± 0.41 mm/mm², ^###^*P* < 0.001) exhibited a marked reduction in neurite outgrowth and length compared to the control (Ctrl, 9.82 ± 0.87 mm/mm²) group. However, Trp treatment restored the neurite outgrowth and length in a dose-dependent manner. Specifically, the neurite length increased from 6.97 ± 0.15 to 7.06 ± 0.79 mm/mm² (^$$$^*P* < 0.001) with Trp treatment, compared to the MGO-induced group (Fig. 7D). Furthermore, microscopic imaging of neurite outgrowth revealed that Trp treatment effectively protected against neuronal cell death by attenuating inflammation-induced damage.

## Discussion

Recent advances in neurogastroenterology have increasingly highlighted the role of gut microbiota in modulating neurodevelopment, neuroinflammation, and the progression of neurodegenerative diseases [28, 29]. Dysbiosis, defined as a disruption in the composition and function of the gut microbiota, has been implicated in a wide range of neurological and psychiatric disorders, including depression, autism spectrum disorders, and AD [2]. These observations have intensified interest in identifying beneficial microbial strains and metabolites that restore gut–brain axis homeostasis and confer neuroprotection. *B. longum*, a well-characterized psychobiotic, has demonstrated the ability to modulate immune responses, attenuate stress, and regulate neurotransmitter-associated pathways [30-32]. Building upon this foundation, the present study investigated the neuroprotective potential of *B. longum* Z1 in an *in vitro* model of MGO-induced glycotoxicity using N2a neuronal cells.

MGO is a highly reactive dicarbonyl compound and a key precursor to AGEs, which accumulate in tissues under metabolic stress. Elevated MGO levels are a hallmark of diabetes and have also been implicated in neurodegenerative diseases such as AD [9, 33]. In our model, MGO exposure led to impaired cell viability, increased ROS and LDH release, and morphological alterations in N2a cells (Fig. 1 and S1). Additionally, *B. longum* Z1 effectively promoted the breakdown of MGO-AGEs, as evidenced by increased free amine production in a concentration-dependent manner (Fig. S2) with an efficacy comparable to that of AG, an AGEs inhibitor. This result supports the hypothesis that *B. longum* Z1 prevents MGO-AGE accumulation, which is associated with memory impairment and cognitive decline.

Mechanistically, MGO triggered the activation of MAPK pathways (p38, JNK, and ERK) and the NF-κB signaling cascade, leading to phosphorylation of downstream effectors and nuclear translocation of NF-κB. These pathways are known to promote the transcription of pro-inflammatory and pro-apoptotic genes and thereby exacerbate neurotoxicity [34, 35]. Notably, *B. longum* Z1 inhibited MAPK phosphorylation and suppressed NF-κB activation, indicating interference with upstream signaling events involved in glycation-induced stress (Fig. 3). In parallel, MGO activated intrinsic apoptotic pathways, as evidenced by increased expression of Bax and cleaved caspase-3 and reduced Bcl-2 levels. *B. longum* Z1 dose-dependently reversed these changes, restoring Bax/Bcl-2 balance and reducing caspase-3 activation, thereby supporting mitochondrial integrity and neuronal survival (Fig. 2).

Interestingly, MGO (data not shown) did not stimulate the production of classical pro-inflammatory cytokines (*e.g.*, IL-1β, IL-6, and TNF-α) or NO production, suggesting that its neurotoxic effects are primarily mediated by non-inflammatory mechanisms. In contrast, LPS robustly induced cytokine and NO production and was therefore used as a positive control to evaluate the anti-inflammatory potential of *B. longum* Z1. Notably, *B. longum* Z1 significantly suppressed LPS-induced IL-1β, IL-6, TNF-α, and NO levels in a concentration-dependent manner, confirming its anti-inflammatory efficacy in a classical neuroinflammatory model (Fig. 4).

Another critical neuroprotective mechanism of *B. longum* Z1 was the enhancement of MGO detoxification. This process is driven primarily by the glyoxalase system, with glyoxalase-1 serving as the rate-limiting enzyme that converts MGO into non-toxic intermediates, thereby preventing AGE formation [36]. Our findings showed that MGO significantly downregulated both the expression and activity of glyoxalase-1 and -2, compromising the cellular detoxification response (Fig. 5). In contrast, *B. longum* Z1 restored their expression and enzymatic function, suggesting its capacity to reduce intracellular MGO accumulation and limit glycation stress. These effects were further supported by reactivation of the Nrf2/Keap1/HO-1 pathway, which plays a central role in maintaining redox homeostasis and directly regulates glyoxalase-1 transcription [37-40] (Fig. 5). Furthermore, *B. longum* Z1 significantly increased glyoxalase-1 enzymatic activity in both the culture medium and cell lysates of MGO-treated N2a cells (Fig. S3). These findings indicate that *B. longum* Z1 not only promotes glyoxalase expression but also boosts glyoxalase-1 activity, potentially reducing intracellular MGO accumulation and mitigating glycation-induced cellular damage.

Metabolomic profiling using UHPLC-Orbitrap-MS/MS identified 47 metabolites, with tryptophan (Trp) emerging as a major bioactive component (Table 1 and Fig. S4). Trp is not only a precursor of serotonin and kynurenine but also plays a vital role in regulating redox balance and immune function [41, 42]. Our data showed that Trp had strong binding affinity for MGO and effectively reduced MGO-induced cytotoxicity in N2a cells (Figs. 6 and 7). Trp restored apoptotic balance (Bax/Bcl-2), suppressed caspase-3 activation, and promoted neurite outgrowth, indicating both cytoprotective and neuroregenerative effects (Fig. 7). Moreover, Trp promoted neurite outgrowth in MGO-challenged N2a cells, suggesting enhancement of neuronal plasticity. These findings are consistent with clinical observations linking low Trp availability to depression and cognitive decline [50, 51], and support the notion that Trp may be a key effector metabolite underlying the neuroprotective actions of *B. longum* Z1.

In addition to *B. longum* Z1, other probiotic strains such as *Lactobacillus plantarum* and *Bifidobacterium infantis* regulate tryptophan (Trp) metabolism through pathways including serotonin synthesis, kynurenine flux, and the production of anti-inflammatory and antioxidant metabolites that influence central nervous system function [43, 44]. The neuroprotective effects observed in this study should be interpreted in the context of these shared metabolic characteristics. To further clarify the specific contribution of Trp to protection against MGO-induced neurotoxicity, comparative studies using *Bifidobacterium* strains with differing Trp biosynthetic capacities will be essential.

This study has several limitations. It was conducted using a single *in vitro* neuronal model (N2a cells), which does not capture the full complexity of the gut–brain axis. Only intracellular metabolites were analyzed, even though Trp and other small molecules are secreted during bacterial growth. Profiling extracellular metabolites under live culture conditions would provide a more accurate representation of the functional output of active probiotics. In addition, the roles of other microbial metabolites were not investigated. Further validation in animal models including germ-free or humanized microbiota systems and in clinical trials is essential to establish the systemic relevance and therapeutic potential of *B. longum* Z1.

In conclusion, this study demonstrates that *B. longum* Z1 protects against MGO-induced neurotoxicity by attenuating MAPK/NF-κB activation, inhibiting mitochondrial apoptosis, enhancing glyoxalase-mediated detoxification, and restoring antioxidant defense mechanisms. The identification of Trp as a functional metabolite further supports its role in mediating gut–brain axis signaling and neuroprotection. These findings highlight the potential of *B. longum* Z1 as a psychobiotic candidate for mitigating glycation-associated neurodegenerative processes and provide a foundation for future exploration of microbe-derived metabolites in brain health.

## Supplemental Materials

Supplementary data for this paper are available on-line only at http://jmb.or.kr.



## Figures and Tables

**Table 1:** Identified metabolites and corresponding peak areas in *B. longum* Z1 as determined by UHPLC-Orbitrap-MS/MS analysis.