# Supplementary material for: Mitigation of Bifidobacterium longum Z1 on Methylglyoxal-Induced Glycotoxicity in Neuron-2A Cells by Enhancing Detoxification Pathways
Source: J Microbiol Biotechnol. 2025 Sep 11;35:e2504018. doi: 10.4014/jmb.2504.04018 (PMC12438956; doi:10.4014/jmb.2504.04018)
Supplement: Supplementary file 1 [file jmb-35-e2504018-supple.pdf]

J. Microbiol. Biotechnol. 2025. 35: e2504018

https://doi.org/10.4014/jmb.2504.04018

Supplementary Figures

Mitigation of *Bifidobacterium longum* Z1 on Methylglyoxal-Induced Glycotoxicity in Neuron-2A Cells by Enhancing Detoxification Pathways

<Supplementary methods>

Cell viability and Morphological Changes

Cell viability was assessed using the MTT [(3-(4,5-dimethylthiazol-2-yl)-2,5-diphenyltetrazolium bromide)] assay. N2a cells (2×10^4 cells/mL) were seeded into 96-well plates, pre-treated with *B. longum* Z1 (1, 5, and 10 µg/mL) or aminoguanidine (AG, 1 mM) for 1 h, followed by treatment with (without) 500 µM MGO for 24 h. Cells were then incubated with 0.5 mg/mL MTT solution for 1 h, the solution was removed, and 200 µL dimethyl sulfoxide (DMSO) was added. The absorbance at 570 nm was measured using a Bio-Rad plate reader (Bio-Rad, USA) (Fig. S1A and S1B). Morphological changes in the mesangial cells were investigated using an IncuCyte Zoom imaging system (Essen Bioscience, USA) (Fig. S1C).

Preparation of Methylglyoxal (MGO)-Derived Advanced Glycation End Products (MGO-AGEs)

For the preparation of MGO-AGEs, 10 mM MGO was reacted with 5 mg/mL of bovine serum albumin and 0.02% sodium azide, and dissolved in PBS (pH 7.4) for 1 week at 37°C. The reacted solution was evaporated, filtered, and dialyzed using a ZebaTM Spin Desalting Column (7 K MWCO; Thermo Fisher Scientific, USA). The filtered GO-AGEs were stored in a freeze-dryer until use.

MGO-AGEs Breaker Assay

The MGO-AGE breaker assay was performed using a derivative of 2,4,6-trinitrobenzene sulfonic acid (TNBSA) for measuring effects,[1] according to a previous study, with minor modifications. MGO-AGEs (0.5 mg/mL) were mixed with corresponding samples and reacted for 1 d at 37°C in the dark. TNBSA within sodium bicarbonate was added to mixed samples, and then incubated once again for 2 h at 37°C. The reactions were stopped by adding a 10% sodium dodecyl sulfate solution and 1 N hydrochloric acid. Free amines were quantified at 340 nm using a microplate reader (Molecular Devices).

Glyoxalase-1 Enzyme Activity Assay

Glyoxalase-1 activity was assessed using a previously described spectrophotometric method with minor modifications [2]. Briefly, N2a cells (2×10^4 cells/mL) were seeded into 96-well plates, pre-treated with *B. longum* Z1 (1, 5, and 10 μg/mL) or aminoguanidine (AG, 1 mM) for 1 h, followed by treatment with (without) 500 μM MGO for 24 h. The conditioned medium and harvested N2a cells were analyzed using the QuantiChrom Glyoxalase I Assay Kit (Shanghai Universal Biotech Co., China). For the lysates of N2a cells, protein concentrations were estimated using the Bradford method [3].

Metabolites Analysis

To extract the intracellular metabolites, 50 mg of *B. longum* Z1 was suspended in 1mL of pure methanol. The samples were homogenized for 10 min using a mixer mill, followed by sonication for 10 min at room temperature. After centrifugation at 15,000 × *g* for 10 min at 4°C, the supernatant was carefully collected and concentrated using a speed vacuum. The dried extract was reconstituted in 100% methanol to achieve a final concentration of 4 mg/mL. A 100 μL aliquot was transferred into a vial for subsequent mass spectrometry analysis. Metabolite profiling was conducted using a Vanquish binary pump C system (Thermo Fisher Scientific) coupled with a Waters ACQUITY UPLC HSS T3 column (150 mm × 2.1 mm, 1.8 μm particle size; Waters) and an Orbitrap Exploris™ 120 mass spectrometer (Thermo Fisher Scientific), following the method described by Na *et al.*, [4].

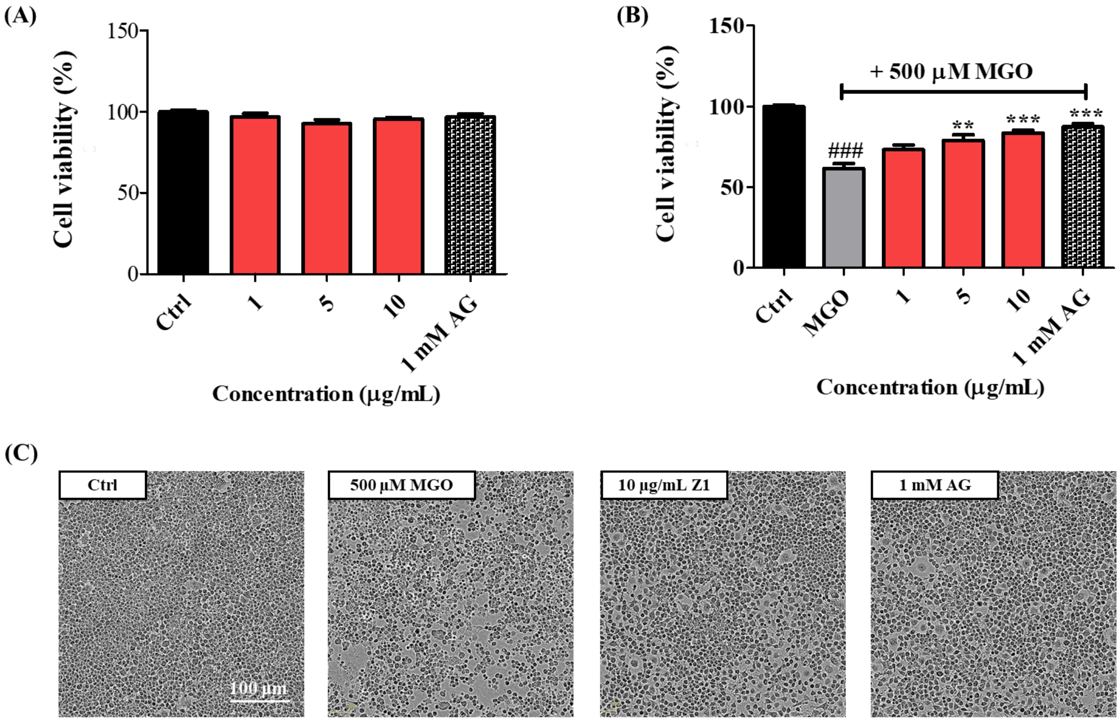

Fig. S1. Effects of *B. longum* Z1 on MGO-induced glucotoxicity in N2a cells. (A, B) Cell viability in N2a cells pre-treated with *B. longum* Z1 (1, 5, and 10 µg/mL) or 1 mM aminoguanidine (AG) for 1 h, followed by MGO (500 µM) exposure (or not) for 24 h. **(C)** Representative photographs of *B. longum* Z1 (1, 5, and 10 µg/mL) pre-treatment protection against MGO-induced cytotoxicity. Scale bar indicates 500 µm. Data are expressed as mean ± SEM (n = 3). $^{###}P < 0.001$ vs. Control (Ctrl) group. $^{\$\$}P < 0.01$ and $^{\$\$\$}P < 0.001$ vs. MGO-induced (MGO) group.

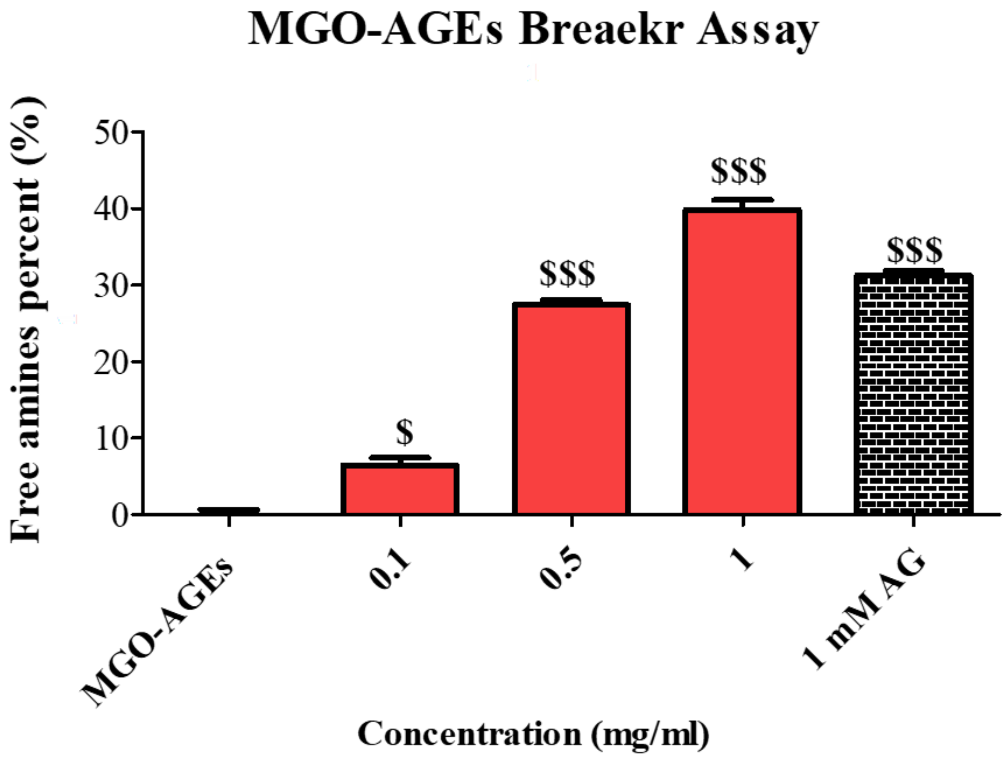

Fig. S2. Effects of *B. longum* Z1 on MGO-AGEs breaker assay. MGO-AGEs (0.5 mg/mL) were incubated with *B. longum* Z1 (100, 500, and 1000 µg/mL) or 1 mM aminoguanidine (AG) at 37 °C for 24 h in the dark. 2,4,6-trinitrobenzene sulfonic acid (TNBSA) in sodium bicarbonate buffer was then added to the reaction mixture, followed by incubation for 2 h at 37 °C. The reaction was terminated with 10% SDS and 1 N HCl. The amounts of free amines released indicative of MGO-AGEs breaking activity was quantified by measuring absorbance at 340 nm using a microplate reader. Data are expressed as mean ± SEM (n = 3). $^{\$}P < 0.05$ and $^{\$\$\$}P < 0.001$ vs. MGO-AGEs group.

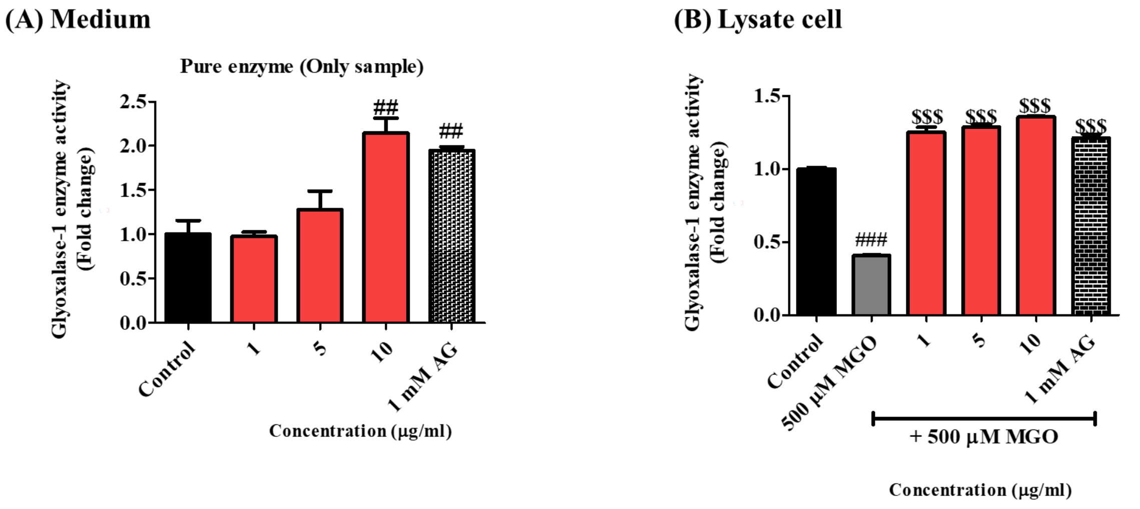

Fig. S3. Effects of *B. longum* Z1 on glyoxalase-1 enzyme activity. Glyoxalase-1 enzyme activity in N2a cells pre-treated with *B. longum* Z1 (1, 5, and 10 μg/mL) or 1 mM aminoguanidine (AG) for 1 h, followed by MGO (500 μM) exposure (or not) for 24 h. **(A)** Glyoxalase-1 enzyme activity from the conditioned medium. **(B)** Glyoxalase-1 enzyme activity from lysate N2a cell. Data are expressed as mean ± SEM (n = 3). $^{\#\#}P < 0.01$ and $^{\#\#\#}P < 0.001$ vs. Control (Ctrl) group. $^{\$\$\$}P < 0.001$ vs. MGO-induced (MGO) group.

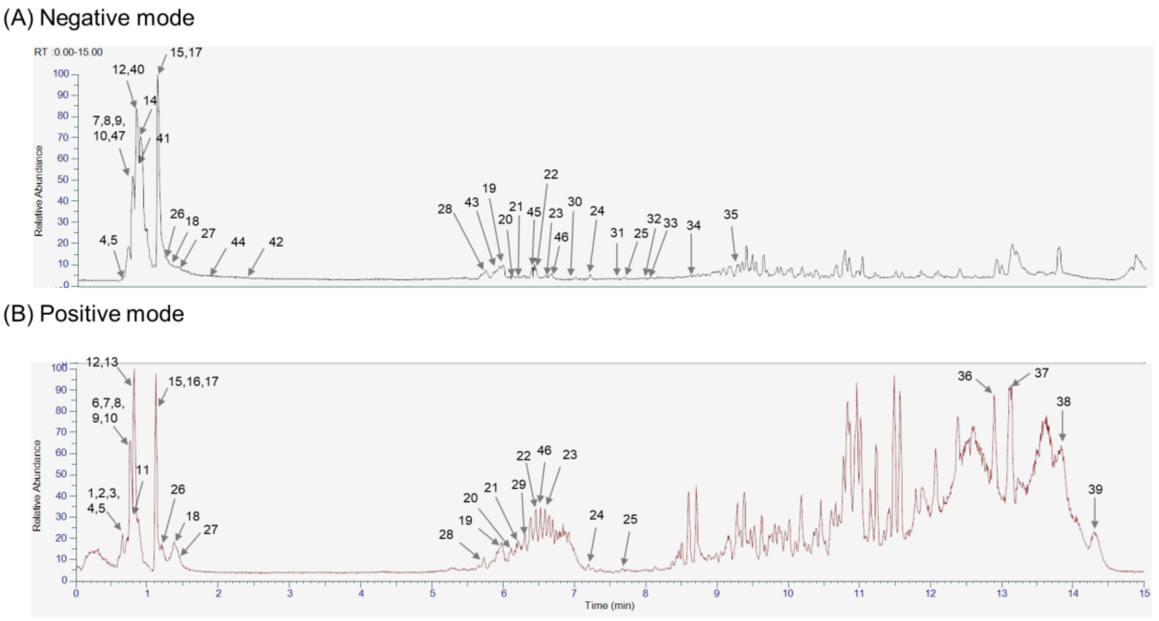

Fig. S4. Chromatograms of the metabolites from *B. longum* Z1 at negative mode (A) and positive mode (B).

References

1. Samsuzzaman M, Lee JH, Moon H, Lee J, Lee H, Lim Y*, et al.* 2022. Identification of a potent NAFLD drug candidate for controlling T2DM-mediated inflammation and secondary damage in vitro and in vivo. *Front. Pharmacol.* **13:** 943879.
2. Angeloni C, Malaguti M, Rizzo B, Barbalace MC, Fabbri D, Hrelia S. 2015. Neuroprotective effect of sulforaphane against methylglyoxal cytotoxicity. *Chem. Res. Toxicol.* **28:** 1234-1245.
3. Bradford MM. 1976. A rapid and sensitive method for the quantitation of microgram quantities of protein utilizing the principle of protein-dye binding. *Anal. Bochem.* **72:** 248-254.
4. Na HH, Kim S, Kim JS, Lee S, Kim Y, Kim SH*, et al.* 2024. Facemask acne attenuation through modulation of indirect microbiome interactions. *NPJ Biofilms Microbiomes* **10:** 50.

<Western blotting assay_raw images>

Fig. 2.

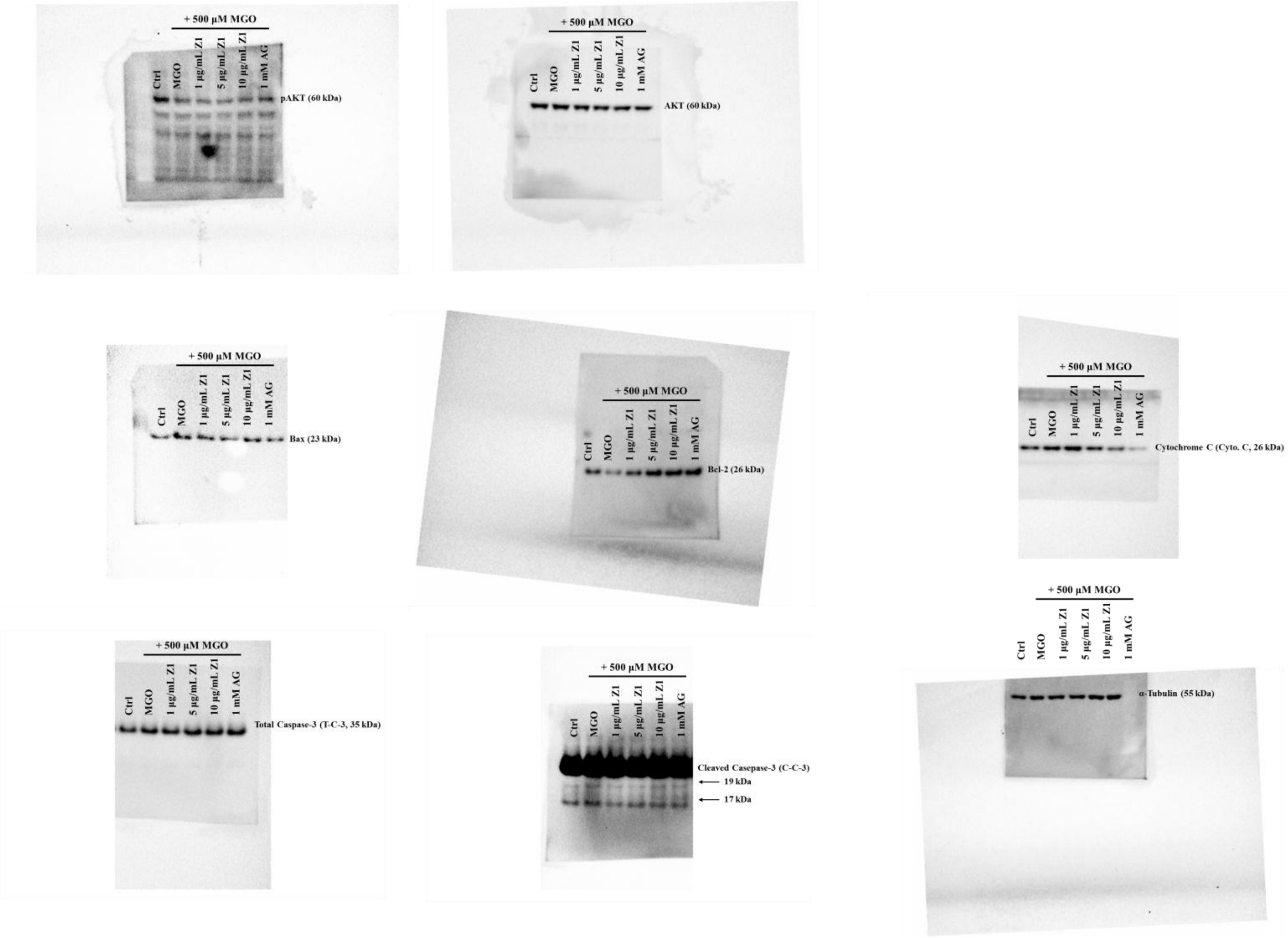

Fig. 3.

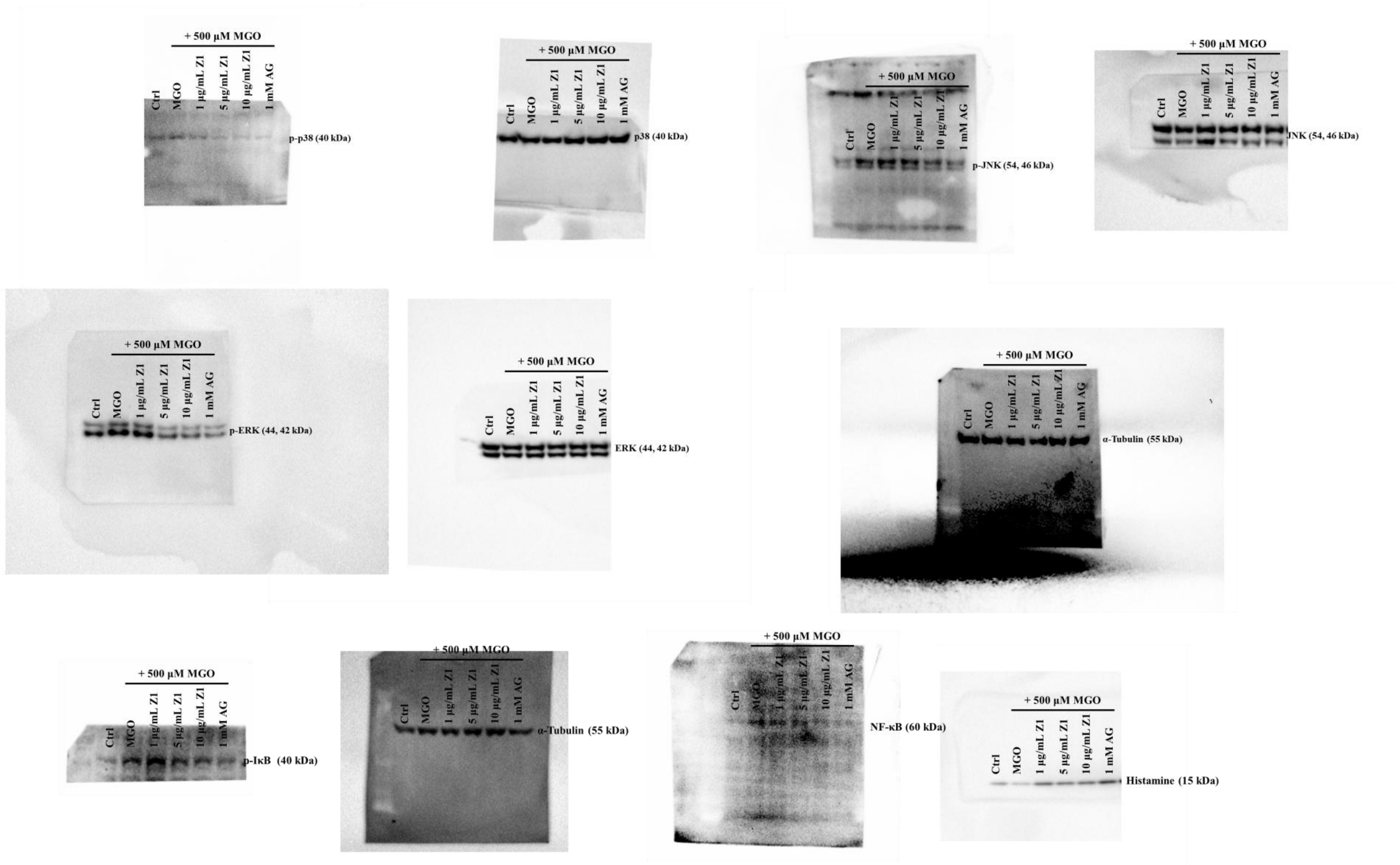
+ 500 μM MGO
Ctrl
MGO
1 μg/mL Z1
5 μg/mL Z1
10 μg/mL Z1
1 mM AG
p-p38 (40 kDa)
p38 (40 kDa)
p-JNK (54, 46 kDa)
JNK (54, 46 kDa)
p-ERK (44, 42 kDa)
ERK (44, 42 kDa)
α-Tubulin (55 kDa)
p-IκB (40 kDa)
NF-κB (60 kDa)
Histamine (15 kDa)

Fig. 4.

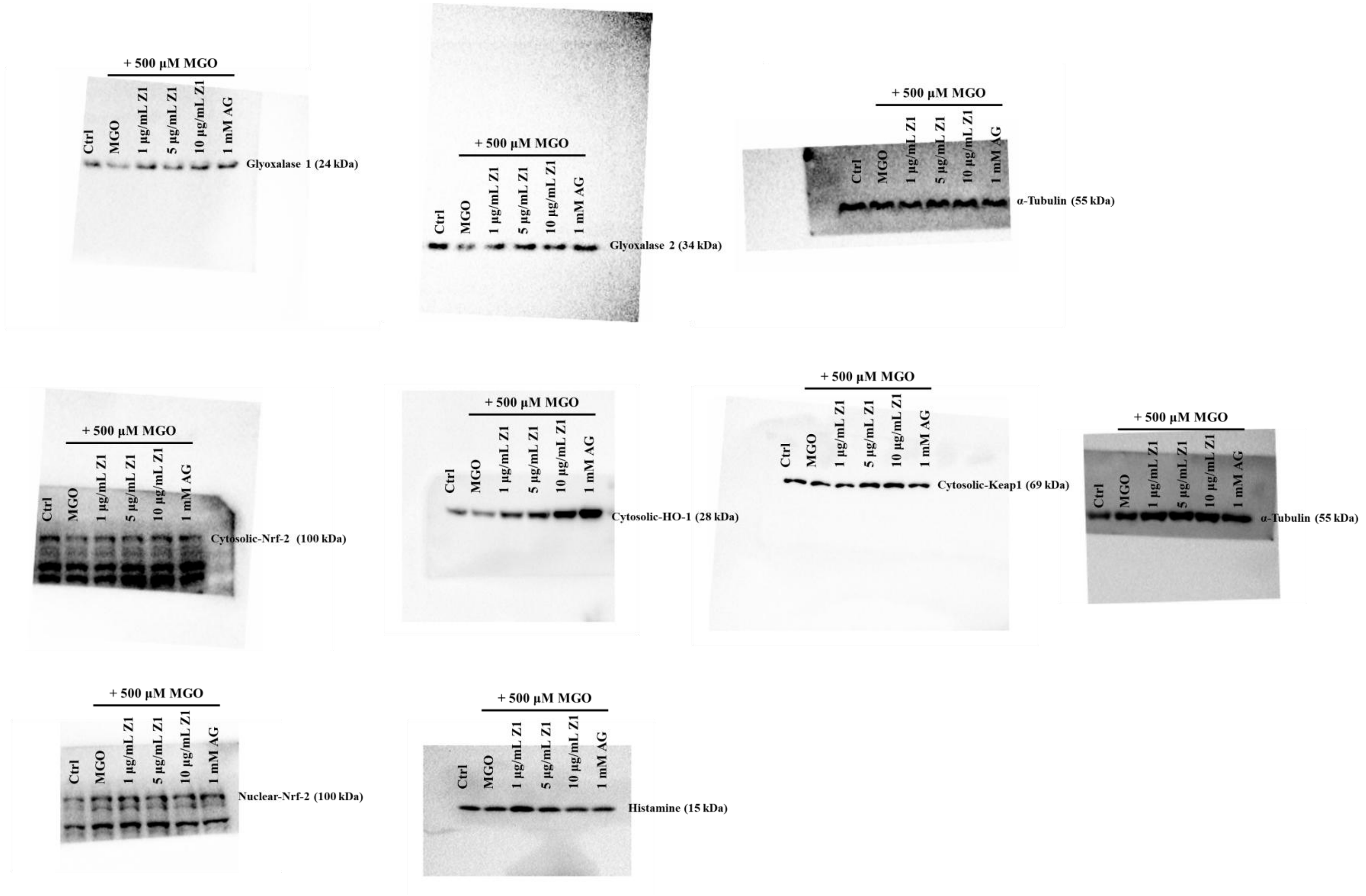
+ 500 μM MGO
Ctrl
MGO
1 μg/mL Z1
5 μg/mL Z1
10 μg/mL Z1
1 mM AG
Glyoxalase 1 (24 kDa)
+ 500 μM MGO
Ctrl
MGO
1 μg/mL Z1
5 μg/mL Z1
10 μg/mL Z1
1 mM AG
Glyoxalase 2 (34 kDa)
+ 500 μM MGO
Ctrl
MGO
1 μg/mL Z1
5 μg/mL Z1
10 μg/mL Z1
1 mM AG
α-Tubulin (55 kDa)
+ 500 μM MGO
Ctrl
MGO
1 μg/mL Z1
5 μg/mL Z1
10 μg/mL Z1
1 mM AG
Cytosolic-Nrf-2 (100 kDa)
+ 500 μM MGO
Ctrl
MGO
1 μg/mL Z1
5 μg/mL Z1
10 μg/mL Z1
1 mM AG
Cytosolic-HO-1 (28 kDa)
+ 500 μM MGO
Ctrl
MGO
1 μg/mL Z1
5 μg/mL Z1
10 μg/mL Z1
1 mM AG
Cytosolic-Keap1 (69 kDa)
+ 500 μM MGO
Ctrl
MGO
1 μg/mL Z1
5 μg/mL Z1
10 μg/mL Z1
1 mM AG
α-Tubulin (55 kDa)
+ 500 μM MGO
Ctrl
MGO
1 μg/mL Z1
5 μg/mL Z1
10 μg/mL Z1
1 mM AG
Nuclear-Nrf-2 (100 kDa)
+ 500 μM MGO
Ctrl
MGO
1 μg/mL Z1
5 μg/mL Z1
10 μg/mL Z1
1 mM AG
Histamine (15 kDa)

Fig. 6.

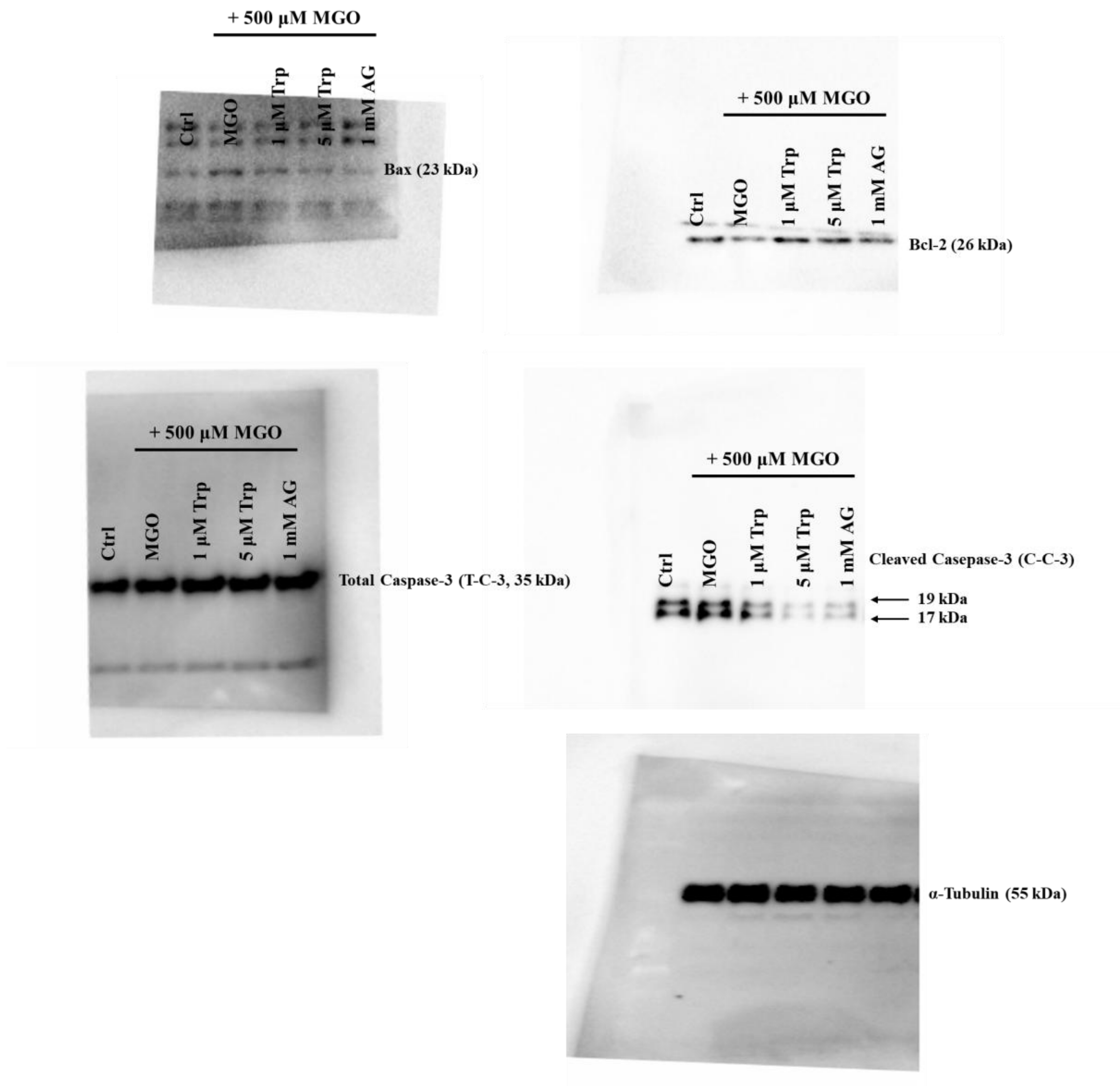
+ 500 µM MGO
Ctrl
MGO
1 µM Trp
5 µM Trp
1 mM AG
Bax (23 kDa)
+ 500 µM MGO
Ctrl
MGO
1 µM Trp
5 µM Trp
1 mM AG
Bcl-2 (26 kDa)
+ 500 µM MGO
Ctrl
MGO
1 µM Trp
5 µM Trp
1 mM AG
Total Caspase-3 (T-C-3, 35 kDa)
+ 500 µM MGO
Ctrl
MGO
1 µM Trp
5 µM Trp
1 mM AG
Cleaved Casepase-3 (C-C-3)
19 kDa
17 kDa
α-Tubulin (55 kDa)
